# STUDENT EXPERIENCES TEACHING AN INTERPROFESSIONAL COMMUNITY-BASED HEALTH PROMOTION PROGRAM FOR OLDER ADULTS

**DOI:** 10.1093/geroni/igad104.2077

**Published:** 2023-12-21

**Authors:** Allexis Mahanna, Marin Livingston, Cara Chapman, Britteny Howell

**Affiliations:** University of Alaska Anchorage, Anchorage, Alaska, United States; University of Alaska Anchorage, Anchorage, Alaska, United States; University of Alaska Anchorage, Anchorage, Alaska, United States; University of Alaska Anchorage, Anchorage, Alaska, United States

## Abstract

Nine (N=9) undergraduate and graduate students collaborated on an interprofessional health promotion program in Spring 2023. Funded by the National Institute on Aging, this student-led program utilized Persuasive Hope Theory with older adult participants to increase their fruit and vegetable intake and exercise patterns in 1-hour weekly sessions. At four community locations for 15 weeks, students gained skills in team-teaching, facilitating group discussions and activities, and engaging in physical activity instruction with older adult participants. At the end of the program, students completed a modified Undergraduate Research Student Self-Assessment (URSSA). As a result of participating in this project, students report understanding and familiarity with the research process, following scientific protocols, problem-solving, and working collaboratively. Students also reported their motivations for joining a research study, satisfaction with working relationships with faculty members and opportunities to present their work, and how these experiences with gerontological research have influenced their career goals. The presenters of this paper are the students themselves, who will lend personal experiences and stories to these survey results. The factors contributing to successful student engagement in this project can be applied by other researchers working in community-based contexts.

